# Anti-Inflammatory and Anticancer Effects of Kaurenoic Acid in Overcoming Radioresistance in Breast Cancer Radiotherapy

**DOI:** 10.3390/nu16244320

**Published:** 2024-12-14

**Authors:** Tae Woo Kim, Seong-Gyu Ko

**Affiliations:** 1 Department of Biopharmaceutical Engineering, Dongguk University-WISE, Gyeongju, Gyeongbuk 38066, Republic of Korea; 2 Department of Preventive Medicine, College of Korean Medicine, Kyung Hee University, Seoul 02447, Republic of Korea; epiko@khu.ac.kr

**Keywords:** kaurenoic acid, NOX4, ROS, apoptosis, ER stress

## Abstract

**Background/Objectives**: Peroxisome proliferator–activated receptor γ (PPARγ) plays a key role in mediating anti-inflammatory and anticancer effects in the tumor microenvironment. Kaurenoic acid (KA), a diterpene compound isolated from *Sphagneticola trilobata* (L.) Pruski, has been demonstrated to exert anti-inflammatory, anticancer, and antihuman immunodeficiency virus effects. **Methods**: In this study, we identified KA as a novel activator of PPARγ with potent anti-inflammatory and antitumor effects both in vitro and in vivo. Given the potential of PPARγ regulators in overcoming radioresistance and chemoresistance in cancer therapies, we hypothesized that KA may enhance the efficacy of breast cancer radiotherapy. **Results**: In a lipopolysaccharide (LPS)-induced mouse inflammation model, KA treatment reduced the levels of pro-inflammatory cytokines, including COX-2, IL-6, IL-1β, and TNFα. In a xenograft mouse mode of breast cancer, KA treatment inhibited tumor growth. Specifically, KA treatment enhanced caspase-3 activity and cytotoxicity against MDA-MB-231 and MCF-7 breast cancer cells. When KA was co-treated with a caspase inhibitor, Z-VAD-FMK, caspase-dependent apoptosis was suppressed in these cells. KA was found to induce the generation of cytosolic calcium ions (Ca^2+^) and reactive oxygen species (ROS), triggering endoplasmic reticulum (ER) stress via the PERK-ATF4-CHOP axis. Hence, the ER stressor thapsigargin (TG) synergized with KA treatment to enhance apoptosis in these cells, while the loss of the PERK or CHOP function inhibited this phenomenon. KA treatment was shown to induce oxidative stress via the NADPH oxidase 4 (NOX4) and stimulate ROS production. Specifically, NOX4 knockdown (KD) and antioxidant treatment (N-acetyl cysteine or diphenyleneiodonium) suppressed such ER stress–mediated apoptosis by inhibiting KA-enhanced caspase-3 activity, cytotoxicity, and intracellular ROS production in the treated cells. In radioresistant MDA-MB-231R and MCF-7R cells, KA combined with 2 Gy radiation overcame radioresistance by upregulating PPARγ and modulating epithelial–mesenchymal transition (EMT) markers, such as E-cadherin, N-cadherin, and vimentin. In PPARγ KD MDA-MB-231R and MCF-7R cells, this phenomenon was inhibited due to reduced PPARγ and NOX4 expression. **Conclusions**: In conclusion, these findings demonstrated KA as a novel PPARγ regulator with promising potential to enhance the efficacy of breast cancer radiotherapy.

## 1. Introduction

Breast cancer is one of the most common cancer types and the leading cause of mortality among women globally. Its incidence is continuing to rise significantly [1,2]. The conventional treatment options for breast cancer include surgical resection, radiotherapy, hormone therapy, immunotherapy, chemotherapy, and targeted therapies [3]. Radiotherapy is considered the most powerful treatment option for breast cancer, but its efficacy is often hampered by radioresistance [4]. Identifying a novel drug that can sensitize tumors to radiotherapy has the potential to overcome this challenge.

Radiotherapy utilizes high-energy radiation from various sources like X-rays, gamma rays, and protons to destroy cancer cells [5]. In the tumor microenvironment (TME), radioresistance is driven by factors such as the DNA damage repair system, genetic alterations, and the emergence of cancer stem-like cells [6]. Various molecular mechanisms contribute to these factors, such as altered epithelial–mesenchymal transition (EMT) markers, the activation of the mTOR-PI3K axis, and changes in cell cycle regulation [7,8]. Combination therapy is effective for overcoming radioresistance and improving the efficacy of cancer radiotherapy [9]. For example, chemotherapy is often combined with radiotherapy in a therapeutic strategy known as “chemoradiation” [10]. Radiotherapy preferentially destroys oxygenated tumor cells and, as a result, tumor cells in a hypoxic TME contribute to the development of radioresistance [11]. In the TME of various tumor types, hypoxic inducible factor-1 (HIF-1) is upregulated. Combining radiation with the HIF-1 inhibitor acriflavine has provided new insights into enhancing the efficacy of cancer radiotherapy [12]. PPARγ is a tumor-suppressing transcription factor with various functional roles, including regulating cell proliferation, apoptosis, and adipose differentiation [13]. In a hypoxic TME, activated PPARγ exerts anticancer effects and overcomes associated resistance by downregulating HIF-1α [14]. PPARγ agonists such as ciglitazone, rosiglitazone, and thiazolidinediones inhibit tumor growth, angiogenesis, and metastasis. They mediate tumor suppression and lipid differentiation by binding to the peroxisome proliferator response element (PPRE) on target gene promoters [15]. PPAR ligands, including rosiglitazone, telmisartan, and pioglitazone, have been shown to overcome resistance in lung cancer cells treated with gefitinib, an epithelial growth factor receptor (EGFR) tyrosine kinase inhibitor [16]. To enhance the sensitivity of radioresistant tumors to radiotherapy, it is essential to deepen our understanding of therapeutic approaches and identify relevant targets in the TME. Recent studies have demonstrated that combining radiation with natural compound–based PPARγ agonists is a powerful strategy to sensitize radioresistant tumor cells [17].

Natural compound–based PPARγ agonists include betaine, curcumin, morusin, piperine, apigenin, and 6-shogaol. Accumulating evidence indicates that these compounds display potent anticancer effects against various cancer types [18]. For example, an apigenin promotes apoptosis and autophagy of cancer cells by downregulating EZH2 in hypoxic gastric cancer [19]. Furthermore, 6-shogaol has been shown to induce ER stress–mediated apoptosis in gefitinib-resistant ovarian cancer cells by modulating EMT markers and upregulating NOX4 [20].

Kaurenoic acid (KA) is a kaurene diterpene compound derived from *Sphagneticola trilobata* (L.) Pruski [21]. KA is found in the leaves of *Copaifera langsdorffii* and its oil-resins. It has various bioactive properties, including anti-inflammatory, anticancer, antioxidant, and antibacterial effects [22]. Recent studies have demonstrated that KA’s anti-inflammatory effect is mediated by the inhibition of prostaglandin E2 (PGE2) and inflammatory cytokines, such as cyclooxygenase-2 (COX-2), nitric oxide synthase (iNOS), and nitric oxide (NO) [23]. KA has also been shown to induce caspase-dependent apoptosis in gastric cancer and cervical cancer cells by promoting DNA damage and modulating cell cycle checkpoint-related markers, including ATM, MYC, CHK2, TP53, CCND1, and BCL2 [24,25].

In the lumen of ER, the disruption of protein folding/refolding and the release of Ca^2+^ induces ER stress responses, leading to the activation of unfolded protein response (UPR) signaling pathways [26]. UPR signaling then activates three UPR sensors: inositol requiring enzyme 1α (IRE1α), PKR-like ER kinase (PERK), and activating transcription factor 6 (ATF6). The stress-inducible ER chaperone protein GRP78 (BIP) dissociates from these sensors to regulate the balance between the three ER transmembrane stress sensor proteins and unfolded proteins [27]. UPRs restore cell homeostasis by mitigating ER stress, but prolonged or excessive activation of UPR pathways can lead to apoptosis [28]. Excessive ROS accumulation, also known as oxidative stress, induces ER stress responses by generating Ca^2+^, leading to apoptosis and mitochondrial dysfunction [29,30]. NADPH oxidases (Noxs), particularly NOX2 and NOX4, contribute significantly to ROS release and associated ER stress signaling pathways [31].

In this study, we investigated KA’s role in regulating PPARγ activity and its anti-inflammatory and anticancer effects mediated by NOX4-induced ER stress signaling pathways and apoptosis in breast cancer. Importantly, we demonstrated KA’s ability to sensitize radioresistant breast cancer cells to radiotherapy.

## 2. Materials and Methods

### 2.1. Reagents

Kaurenoic acid (KA, SMB00177), N-acetylcysteine (NAC), diphenyleneiodonium (DPI), lipopolysaccharide (LPS; L4391), Z-VAD-FMK, and TG (T9033) were purchased from Sigma-Aldrich (St. Louis, MO, USA).

### 2.2. Cell Culture

MCF-10A (normal human breast cells) was purchased from the American Type Culture Collection (ATCC, Manassas, VA, USA) and cultured at 37 °C under 5% CO_2_ in Dulbecco’s Modified Eagle Medium/F-12 (DMEM/F12; Welgene, Gyeongsangbuk-do, Republic of Korea) supplemented with hydrocortisone (0.5 μg/mL; Sigma-Aldrich), 10% fetal bovine serum (FBS; Welgene), insulin (0.01 mg/mL; Sigma-Aldrich), EGF (20 ng/mL; Invitrogen), and 1% penicillin-streptomycin (PS; Welgene). Human breast cancer cell line (MCF-7, SK-BR-3, T47D, HCC-1419, HT-20, and MDA-MB-231) and 3T3-L1 murine adipocytes were purchased from the Korean Cell Line Bank (Seoul, Republic of Korea) and cultured at 37 °C under 5% CO_2_ in DMEM supplemented with 10% FBS and 1% PS.

### 2.3. Cell Viability and Proliferation Assays

To determine the impact of KA on the viability of treated cells, the WST-1 assay (Roche Applied Science, Indianapolis, IN, USA) was conducted in both treatment time- and concentration-dependent manners (KA; 10, 50, 100, 200, and 300 µM; 24 h). Breast cancer cells were seeded and cultured in a 96-well cell culture plate (1 × 10^4^ cells/well). Following the manufacturer’s protocol, the absorbance at 450 nm was measured from each well using a microplate reader (Molecular Devices, CA, USA).

### 2.4. Lactate Dehydrogenase (LDH) Cytotoxicity Assay

To assess the cytotoxicity of KA against breast cancer cells, LDH cytotoxicity assay (Abcam, Cambridge, MA, USA) was conducted in both treatment time- and concentration-dependent manners. Breast cancer cells were seeded and cultured in a 96-well cell culture plate (1 × 10^4^ cells/well). Following the manufacturer’s protocol, the absorbance at 490 nm was measured from each well using a microplate reader.

### 2.5. Colorimetric Caspase-3 Activity Assay

To assess KA’s impact on caspase-3 activity in breast cancer cells, a colorimetric caspase-3 activity assay (Abcam) was conducted in both treatment time- and concentration-dependent manners. Breast cancer cells were seeded and cultured in a 96-well cell culture plate (1 × 10^4^ cells/well). Following the manufacturer’s protocol, the absorbance at 490 nm was measured from each well using a microplate reader.

### 2.6. Intracellular Ca^2+^ Assay

To assess the level of the intracellular Ca^2+^ in breast cancer cells treated with KA, a cytosolic Ca^2+^ assay (Abcam) was conducted in both treatment time- and concentration-dependent manners. Breast cancer cells were seeded and cultured in a 96-well cell culture plate (1 × 10^4^ cells/well). Following the manufacturer’s protocol, the fluorescence at 575 nm was measured from each well using FilterMax F5 (Molecular Devices, San Jose, CA, USA).

### 2.7. Intracellular ROS Assay

To assess the level of ROS in breast cancer cells treated with KA, an intracellular ROS assay (Abcam) was conducted in both treatment time- and concentration-dependent manners. Breast cancer cells were seeded and cultured in a 96-well cell culture plate (1 × 10^4^ cells/well). Following the manufacturer’s protocol, the fluorescence at 605 nm (excitation at 520 nm) was measured from each well using FilterMax F5.

### 2.8. Radioresistant MDA-MB-231R and MCF-7R Cell Lines

MDA-MB-231 and MCF-7 cells were cultured in 60-mm culture plates. The cells were irradiated at 4 Gy daily for 90 days to establish radioresistant MDA-MB-231R and MCF-7R cell lines.

### 2.9. Radiotherapy

MDA-MB-231, MCF-7, MDA-MB-231R, and MCF-7R cells were cultured in 60-mm culture plates at 37 °C under 5% CO_2_. The cells were irradiated using an irradiator with a cesium-137 source (Atomic Energy of Canada, Ltd., Mississauga, ON, Canada).

### 2.10. Colony-Forming Cell Assays

MDA-MB-231, MCF-7, MDA-MB-231R, and MCF-7R cells were cultured in 60-mm culture plates at 37 °C under 5% CO_2_ for colony formation. The colonies were stained with 0.5% crystal violet solution (Sigma-Aldrich, St. Louis, MO, USA).

### 2.11. Transfection Assay

Small interfering RNAs (siRNAs) for CHOP (Bioneer, Daejeon, Republic of Korea), Nox4 (Santacruz, Dallas, TX, USA), and PERK (Santacruz) were used for gene-silencing experiments. For PPARγ silencing, short hairpin RNA (shRNA) particles (Santa Cruz) were used. MDA-MB-231 and MCF-7 cells were first seeded in 6-well culture plates. Following the manufacturer’s protocol, the cells were transfected with siRNAs (30 nmol/mL) or PPARγ shRNA particles (20 μL). Lipofectamine 2000 (Invitrogen, Carlsbad, CA, USA) was used as the transfection agent for siRNAs. To perform luciferase reporter assay, breast cancer cells MDA-MB-231 and MCF-7 cells and 3T3-L1 murine adipocytes were co-transfected with the pGL3 PPRE-luciferase reporter vector (1.5 µg, Promega) or the pGL3 luciferase reporter vectors (1.5 µg, Promega) using a luciferase assay system (Promega, Madison, WI, USA). Following the manufacturer’s protocol, the luciferase activity at 550 nm was measured from each well using FilterMax F5.

### 2.12. Protein and RNA Purification

Breast cancer cells and 3T3-L1 murine adipocytes were cultured in 100-mm culture plates at 37 °C under 5% CO_2_. Following the manufacturer’s protocol, total RNA and protein were isolated with Trizol reagent (Invitrogen) and extracted using the radio-immunoprecipitation assay (RIPA) lysis buffer (Thermo Fisher Scientific, CA, USA).

### 2.13. Quantitative Reverse Transcription Polymerase Chain Reaction (qRT PCR) and Western Blot Analyses

For qRT-PCR assay, all reactions were performed in triplicates. The primers were adopted from a study by Kim et al. and purchased from Bioneer (Daejeon, Republic of Korea) [20]. To assess relative gene expression levels, the 2^−ΔΔCt^ method was used. To assess protein expression levels, Western blot analysis was performed. After separating the proteins via SDS-PAGE, they were transferred to PVDF membranes, which were blocked with 5% skim milk and incubated with primary antibodies. The primary antibodies used were as follows: cleaved caspase-9 (Cell Signaling, Danvers, MA, USA), CHOP (Cell Signaling), ATF4 (Cell Signaling), p-PERK(Thr980) (Cell Signaling), cleaved caspase-3 (Cell Signaling), p-eIF2α (Ser51) (Cell Signaling), PERK (Cell Signaling), eIF2α (Santa Cruz), GRP78 (Santa Cruz), β-actin (Santa Cruz), PPARγ (Proteintech, Rosamond, Illinois, USA), and Nox4 (Proteintech). HRP-conjugated secondary antibodies were used with anti-rabbit IgG HRP-linked antibody (Santa Cruz) and m-IgGK BP-HRP-linked antibody (Santa Cruz). The membranes were visualized using a chemiluminescent substrate for HRP (MilliporeSigma, Burlington, MA, USA).

### 2.14. Tumor and Inflammation Mouse Models

All mouse models were stabilized using female mice. Five-week-old mice were obtained from OrientBio, Inc. (Daejeon, Republic of Korea) and acclimated in a sterile room for one week on an NIH-7 open formula diet. The mice were then divided randomly into three groups. All mouse experiments were conducted in accordance with the guidelines of the Kyung-Hee University Animal Care and Use Committee. The MDA-MB-231 tumor model was established by subcutaneously injecting 1 × 10^7^ cells suspended in PBS into the right dorsal flanks of athymic BALB/c nude mice (*nu/nu*). When the average tumor volume reached 200 mm^3^, the mice were randomly divided into three groups (*n* = 10): control (PBS), 100 mg/kg KA, and 200 mg/kg KA. The specified doses of KA or PBS were administered by intraperitoneal (i.p.) injection twice weekly. Tumor volume (mm^3^) was calculated using the following formula: (*L* × *W*^2^)/2, where *L* is the length and *W* is the width of the tumor. (mm^3^). The anti-inflammatory effects of KA were assessed using the LPS-induced inflammation model. The mice were randomly divided into three groups: PBS, LPS, and LPS + KA. The groups receiving LPS were administered 20 mg/kg of LPS by i.p. injection. The LPS + KA group was administered with 100 mg/kg of KA by ip injection twice weekly. The survival rate was monitored for 12 days following LPS injection, and blood and tissues were collected for analysis.

### 2.15. Cytokine Levels

Raw264.7 and J774.1 cells were seeded in 96-well culture plates (1 × 10^4^ cells/well). The cells were treated with 1 μg/mL LPS in the absence or presence of KA (0, 25, 50, and 100 μM; 24 h) for 24 h. The protein levels of IL-6, IL-1β, and TNF-α were assessed via enzyme-linked immunosorbent assay (ELISA) following the manufacturer’s protocol: IL-6 (DY-406; R&D Systems), IL-1β (DY-401; R&D Systems), and TNF-α (DY-410; R&D Systems).

### 2.16. Statistical Analysis

All experiments were conducted at least three times. Statistical significance was analyzed using analysis of variance (ANOVA) and Student’s *t*-test, with a *p*-value < 0.05 considered statistically significant.

## 3. Results

### 3.1. KA Modulates PPARγ Activity in 3T3-L1 and Breast Cancer Cells

**Figure 1:**
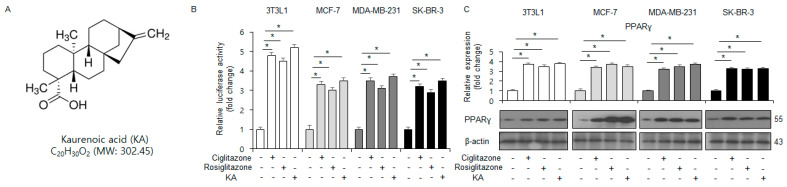
KA-induced enhancement in PPARγ activity of kaurenoic acid (KA). (**A**) The chemical structure of KA. (**B**,**C**) Luciferase activity corresponding to the PPAR response element reporter gene. The mRNA and protein levels in 3T3-L1, MCF-7, MDA-MB-231, and SK-BR-3 cells treated with 100 µM KA, 10 µM ciglitazone, or 20 µM rosiglitazone. *, *p* < 0.05.

The chemical structure of KA is shown in Figure 1A. PPRE luciferase reporter assay, qRT-PCR, and Western blot analyses indicated that a 100 µM KA treatment enhanced PPRE activity and upregulated the protein and mRNA levels of PPARγ in 3T3-L1, MDA-MB-231, SK-BR-3, and MCF-7 cells treated with ciglitazone, rosiglitazone, and KA (Figure 1B,C).

### 3.2. KA Mitigates Inflammation in LPS-Treated Macrophages and Sepsis Mouse Model

**Figure 2:**
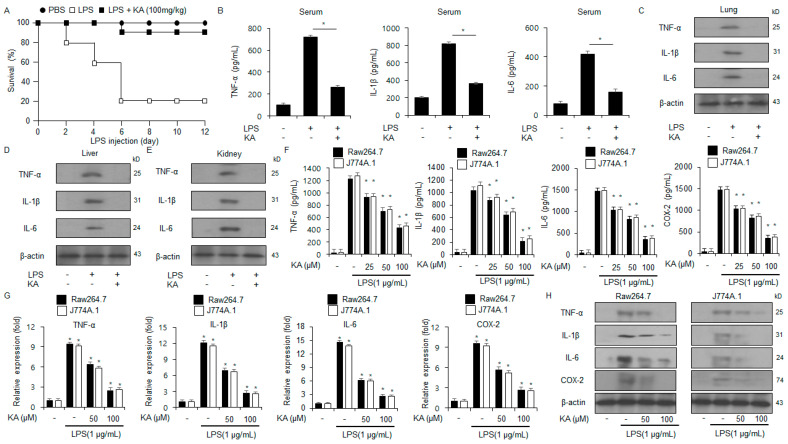
The impact of KA on the mRNA and protein levels of inflammatory cytokines in LPS-treated macrophages. (**A**) C57BL/6 mice were administered with LPS (20 mg/kg) via i.p. injection. The treatment group received KA (100 mg/kg) via i.p. injection. The survival rate of all groups (*n* = 10) was analyzed daily for 12 days following LPS injection. (**B**–**E**) The protein levels of IL-1β, IL-6, and TNF-α in the serum, lungs, liver, and kidneys of the treated mice, as assessed by ELISA and Western blot. (**F**–**H**) The mRNA and protein levels of IL-1β, IL-6, and TNF-α in LPS (1 µg/mL)-treated Raw264.7 and J774.1 cells in the presence or absence of KA (0, 25, 50, and 100 µM; 24 h), as assessed by ELISA, Western blot, and qRT-PCR. β-actin was used to normalize the relative mRNA and protein levels. *, *p* < 0.05. All experiments were conducted three times.

To assess the anti-inflammatory effects of KA, the LPS-induced sepsis mouse model was established. Compared to the LPS group, the LPS + KA group showed approximately a 4-fold enhancement in survival rate (Figure 2A). We next assessed the protein levels of immune regulating cytokines, including IL-6, IL-1β, and TNF-α, via ELISA and Western blot. KA treatment notably reduced the levels of TNF-α, IL-6, and IL-1β in the kidneys, lungs, liver, and serum of the treated mice (Figure 2B–E). Furthermore, LPS-treated macrophages (Raw264.7 and J774.1) were used to confirm the anti-inflammatory effects of KA. As assessed by qRT-PCR, ELISA, and Western blot, KA treatment dramatically downregulated the protein levels of inflammatory cytokines, including COX-2, IL-1β, IL-6, and TNF-α, in a dose-dependent manner (Figure 2F–H). This indicated that KA treatment effectively prevents the LPS-induced production of inflammatory cytokines in macrophages.

### 3.3. KA Induces Caspase-Dependent Apoptosis in Breast Cancer Cells

**Figure 3:**
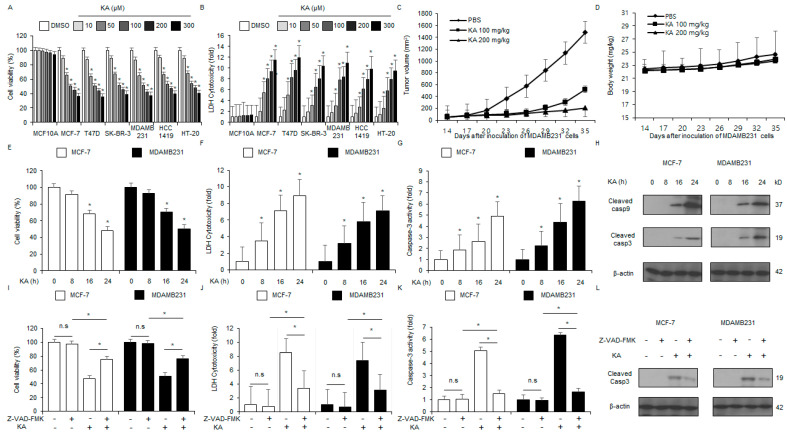
The in vitro and in vivo anticancer effects of KA. (**A**,**B**) LDH and WST-1 assays were conducted for cells (MCF-10A, HT-20, SK-BR-3, T-47D, HCC1419, MDA-MB-231, and MCF-7) treated with varying doses of KA (0, 10, 50, 100, 200, and 300 µM; 24 h). (**C**,**D**) The MDA-MB-231 tumor model was established by injecting 1 × 10^7^ cells into the right dorsal flank of nude mice (*n* = 10 per group). KA (100 and 200 mg/kg) was administered (i.p. injection) twice weekly. The body weights of the treated mice were measured twice weekly. (**E**–**H**) MDA-MB-231 and MCF-7 cells were treated with KA for varying durations (0, 8, 16, and 24 h; 100 µM) and subjected to caspase-3, LDH cytotoxicity, and WST-1 assays. Western blot analysis of cleaved caspase-9 and -3 was also conducted following KA treatment for the indicated durations; *, *p* < 0.05. β-actin was used as the loading control. (**I**–**L**) MCF-7 and MDA-MB-231 cells were pretreated with Z-VAD-FMK (50 μM) for 4 h before KA treatment (100 µM, 24 h). Caspase-3 activity, LDH cytotoxicity, and WST-1 assays were conducted; *, *p* < 0.05. n.s, no significance. Western blot analysis was performed to assess the level of cleaved caspase-3. β-actin was used as the loading control.

The in vitro antitumor effects of KA were assessed against breast cancer or normal cells (SK-BR-3, MCF-7, HT-20, MDA-MB-231, HCC1419, and T-47D or MCF-10A) at varying doses of KA (10, 50, 100, 200, and 300 µM) (Figure 3A,B). The in vivo antitumor efficacy of KA was evaluated using the MDA-MB-231 tumor mouse model. KA treatment (100 mg/kg and 200 mg/kg) significantly inhibited tumor growth compared to the control group (Figure 3C) without causing body weight loss (Figure 3D). The in vitro anticancer effects of KA were investigated in a time-dependent manner (0, 8, 16, and 24 h; 100 µM). MCF-7 and MDA-MB-231 cells were treated with KA and subjected to LDH cytotoxicity, WST-1, and colorimetric caspase-3 activity assays. KA treatment showed enhanced colorimetric caspase-3 activity and cytotoxicity in a time-dependent manner (Figure 3E–G). Western blot analysis also indicated that KA induces time-dependent cleavage of caspase-3 and -9 (Figure 3H). To assess whether KA treatment mediates apoptosis through the caspase-related pathway, MDA-MB-231 and MCF-7 cells were co-treated with Z-VAD-FMK, a cell-permeant pan-caspase inhibitor. At 50 μM KA, the Z-VAD-FMK treatment alone did not significantly affect cytotoxicity, caspase-3 activity, or cell viability. At 100 µM KA, however, cytotoxicity and caspase-3 activity increased. When 50 μM Z-VAD-FMK was co-treated with 100 µM KA, cytotoxicity and colorimetric caspase-3 activity inhibited significantly, leading to an increase in cell viability compared to KA treatment alone (Figure 3I–K). Western blot analysis indicated that this co-treatment reduced the level of cleaved caspase-3 compared to KA treatment alone (Figure 3L). These findings suggested that KA inhibits the proliferation of breast cancer cells via the caspase-dependent signaling pathway.

### 3.4. KA-Induced ER Stress Mediates Apoptosis in Breast Cancer Cells

**Figure 4:**
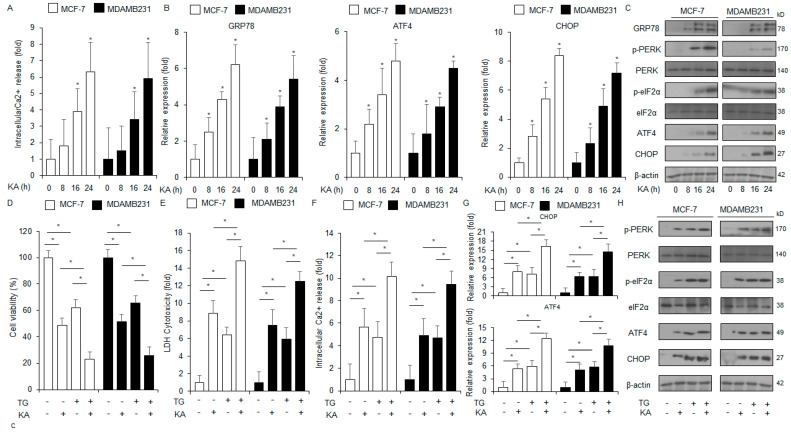
KA promotes the production of intracellular Ca^2+^ and apoptosis. (**A**) MCF-7 and MDA-MB-231 cells were treated with KA for varying durations (0, 8, 16, and 24 h; 100 µM) and subjected to intracellular Ca^2+^ assay; *, *p* < 0.05. (**B**) The mRNA levels of CHOP, ATF4, and GRP78 were assessed by qRT-PCR. β-actin was used as the loading control. (**C**) MDA-MB-231 and MCF-7 cells were treated with KA for varying durations (0, 8, 16, and 24 h; 100 µM). Western blot analysis was conducted for the proteins associated with the ER stress signaling pathway: CHOP, ATF4, GRP78, p-eIF2α, and p-PERK. β-actin was used as the loading control. (**D**–**F**) MCF-7 and MDA-MB-231 cells were treated with 3 μM TG and 100 µM KA for 24 h. LDH cytotoxicity, intracellular Ca^2+^, and cell viability assays were conducted; *, *p* < 0.05. (**G**,**H**) The mRNA levels of CHOP and ATF4 were assessed by qRT-PCR. Western blot analysis was conducted to examine the protein levels of p-eIF2α, p-PERK, CHOP, and ATF4 in MDA-MB-231 and MCF-7 cells treated with 100 µM KA and 3 µM TG for 24 h. β-actin was used as the loading control.

Ca^2+^ regulates immune responses, cell migration, survival, and death [32]. Excessive Ca^2+^ from the ER lumen in the cytosol induces ER stress, leading to apoptosis [33]. Intracellular Ca^2+^ assay revealed that KA treatment promotes Ca^2+^ release in MDA-MB-231 and MCF-7 cells in a time-dependent manner (Figure 4A). Furthermore, qRT-PCR analysis of these cells indicated that KA treatment enhanced the mRNA levels of CHOP, ATF4, and GRP78 in a time-dependent manner; Western blot analysis showed that the protein levels of p-eIF2α, p-PERK, CHOP, ATF4, and GRP78 enhanced with KA treatment (Figure 4B,C). To assess whether ER stress is associated with KA-induced apoptosis, the cells were co-treated with KA and the ER stressor TG. This co-treatment synergistically increased the intracellular Ca^2+^ production and cytotoxicity (Figure 4D–F). Furthermore, the levels of CHOP, ATF4, p-eIF2α, and p-PERK also significantly increased following the treatment (Figure 4G,H).

### 3.5. PERK or CHOP Silencing Inhibits KA-Induced Apoptosis in Breast Cancer Cells

**Figure 5:**
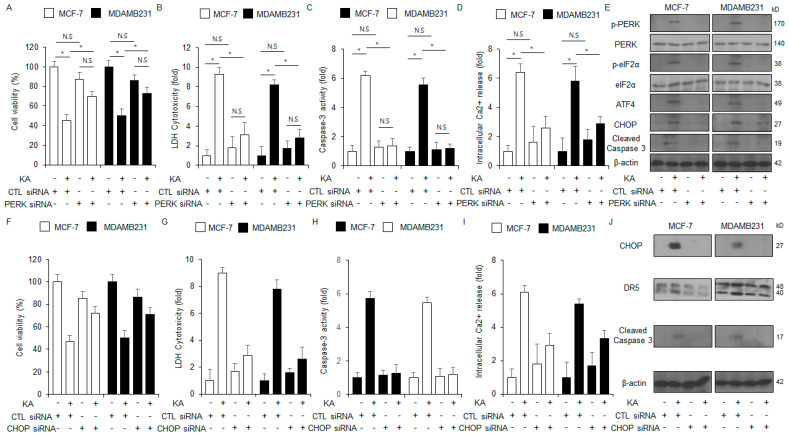
PERK silencing inhibits KA-induced apoptosis in breast cancer cells. (**A**–**E**) PERK siRNA was transfected into MDA-MB-231 and MCF-7 cells. The cells were then treated with 100 µM KA for 24 h. Intracellular Ca^2+^, caspase-3 activity, LDH cytotoxicity, and WST-1 assays were conducted; *, *p* < 0.05. N.S, no significance. Western blot analysis was conducted to assess the protein levels of cleaved caspase-3, CHOP, ATF4, p-eIF2α, and p-PERK in MDA-MB-231 and MCF-7 cells treated with 100 µM KA for 24 h. β-actin was used as the loading control. (**F**–**J**) CHOP siRNA was transfected into MDA-MB-231 and MCF-7 cells. The cells were then treated with 100 µM KA for 24 h. Intracellular Ca^2+^, caspase-3 activity, LDH cytotoxicity, and WST-1 assays were conducted; *, *p* < 0.05. Western blot analysis was conducted to assess the protein levels of cleaved caspase-3, CHOP, and DR5 in MDA-MB-231 and MCF-7 cells treated with 100 µM KA for 24 h. β-actin was used as the loading control.

To investigate whether KA-induced apoptosis is dependent on PERK or CHOP, MDA-MB-231 and MCF-7 cells were transfected with siRNAs for the corresponding genes. PERK silencing reduced KA-induced enhancement in caspase-3 activity, intracellular Ca^2+^ activity, and cytotoxicity (Figure 5A–D). Western blot analysis indicated that p-PERK, p-eIF2α, cleaved caspase-3, ATF4, and CHOP levels were also decreased after PERK silencing in KA-treated MDA-MB-231 and MCF-7 cells (Figure 5E). CHOP silencing also reduced KA-induced enhancements in caspase-3 activity, intracellular Ca^2+^ activity, and cytotoxicity (Figure 5F–I). Western blot analysis indicated that CHOP knockdown reduced the levels of cleaved caspase-3, CHOP, and death receptor 5 (DR5) in KA-treated MDA-MB-231 and MCF-7 cells (Figure 5J). These findings demonstrated that KA-induced apoptosis in breast cancer cells is mediated by ER stress.

### 3.6. Nox4 Silencing Inhibits KA-Induced Apoptosis in Breast Cancer Cells

**Figure 6:**
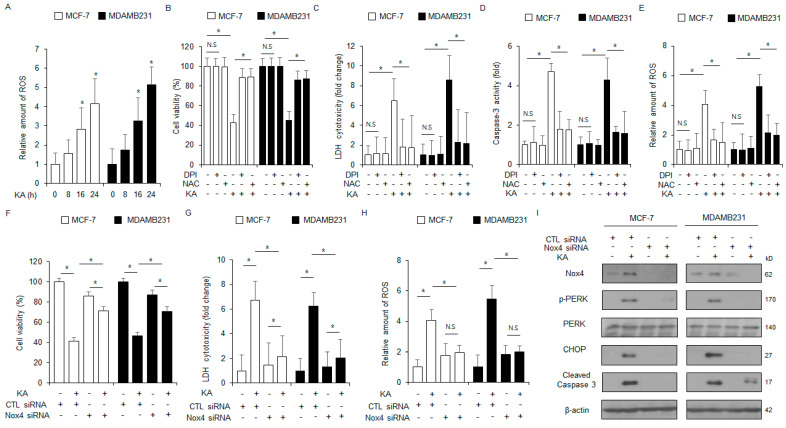
NOX4 silencing inhibits ROS-induced ER stress and apoptosis in KA-treated breast cancer. (**A**) MDA-MB-231 and MCF-7 cells were treated with 100 µM KA for the indicated durations and subjected to intracellular ROS assay DCFDA; *, *p* < 0.05. (**B**–**E**) MDA-MB-231 and MCF-7 cells were treated with 100 µM KA, 1 µM DPI, and 100 µM NAC for 24 h. Caspase-3 activity, LDH cytotoxicity, intracellular ROS, and WST-1 assays were conducted; *, *p* < 0.05. N.S, no significance. (**F**–**I**) NOX4 siRNA was transfected into MDA-MB-231 and MCF-7 cells and then treated with 100 µM KA for 24 h. Intracellular ROS, WST-1, and LDH cytotoxicity assays were conducted; *, *p* < 0.05. N.S, no significance. Western blot analysis was conducted to assess the protein levels of cleaved caspase-3, CHOP, NOX4, PERK, and p-PERK in MDA-MB-231 and MCF-7 cells treated with 100 µM KA for 24 h. β-actin was used as the loading control.

We next assessed the impact of KA treatment on intracellular ROS production. KA treatment enhanced the level of ROS release in breast cancer cells over varying treatment times (Figure 6A). Next, MDA-MB-231 and MCF-7 cells were treated with ROS inhibitors (DPI or NAC) in the presence of KA. This co-treatment KA-induced enhancements in cytotoxicity, intracellular ROS generation, and caspase-3 activity (Figure 6B–E). Next, the cells were transfected with a NOX4-specific siRNA and treated with KA. NOX4 silencing reduced the increases in intracellular ROS and cytotoxicity induced by KA treatment (Figure 6F–H). Western blot analysis indicated that NOX4 silencing also inhibited the enhancements in the levels of CHOP, cleaved caspase-3, p-PERK, and NOX4 (Figure 6I). These findings indicated that NOX4 contributes to KA-induced intracellular ROS release and apoptosis in breast cancer cells.

### 3.7. KA Sensitizes Radioresistant Breast Cancer Cells to Radiotherapy by Modulating EMT Markers

**Figure 7:**
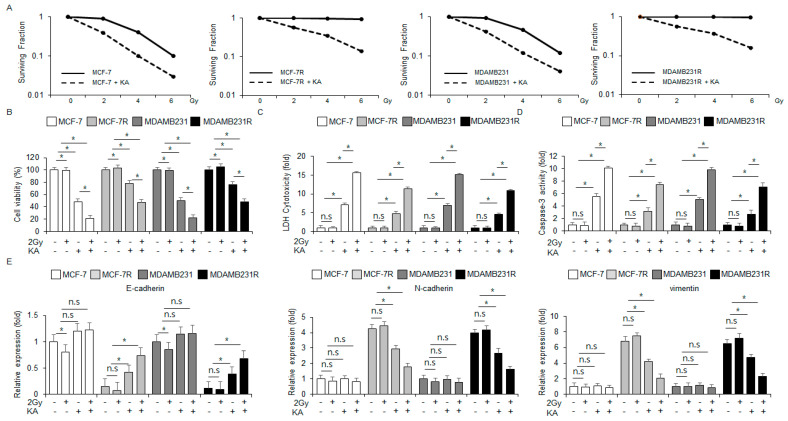
Radiation combined with KA overcomes radioresistance in breast cancer cells. (**A**) Colony formation analysis was performed for MDA-MB-231, MCF-7, MDA-MB-231R, and MCF-7R cells following radiation at varying intensities (0, 2, 4, and 6 Gy) and/or KA treatment. The cell survival rate was quantified; *, *p* < 0.05. (**B**–**D**) MDA-MB-231, MCF-7, MDA-MB-231R, and MCF-7R cells treated with 100 µM KA and 2 Gy radiation for 24 h were subjected to caspase-3 activity, WST-1, and LDH cytotoxicity assays; *, *p* < 0.05. n.s, no significance. (**E**) The mRNA levels of vimentin, N-cadherin, and E-cadherin were assessed by qRT-PCR in MDA-MB-231, MCF-7, MDA-MB-231R, and MCF-7R cells treated with 100 µM KA and 2 Gy radiation for 24 h; *, *p* < 0.05. n.s, no significance. β-actin was used the loading control.

Although radiotherapy is a powerful therapy method to kill breast cancer cells, it frequently mediates radioresistance [34]. Next, we investigated whether KA sensitizes radioresistant MDA-MB-231R and MCF-7R cells using a colony formation assay. KA treatment synergized with varying radiation intensities (2, 4, and 6 Gy) to enhance cytotoxic effects against MDA-MB-231R, MCF-7R, MDA-MB-231, and MCF-7 cells (Figure 7A). In MCF-7 and MDA-MB-231 cells, KA enhanced caspase-3 activity and cytotoxicity. KA combined with 2 Gy radiation further increased these properties and significantly reduced cell viability, with radiation alone having no significant effects (Figure 7B–D). In MCF-7R and MDA-MB-231R cells, KA treatment again enhanced cytotoxicity and caspase-3 activity, leading to synergistic anticancer effects when combined with 2 Gy radiation; radiation alone had no significant effects in these cells (Figure 7B–D). To examine whether this combination modulates the EMT process, the radioresistant cells were subjected to qRT-PCR analysis. Both KA alone and co-treatment with 2 Gy radiation increased the mRNA level of E-cadherin and decreased the mRNA levels of vimentin and N-cadherin in MCF-7R and MDA-MB-231R cells. On the other hands, the nonresistant cells showed no significant changes in these mRNA levels (Figure 7E). These findings indicated that combining KA with radiation may be an effective strategy for treating radioresistant breast cancer.

### 3.8. PPARγ Silencing Inhibits ER Stres–Induced Apoptosis in KA-Treated MCF-7R and MDA-MB-231R Cells

**Figure 8:**
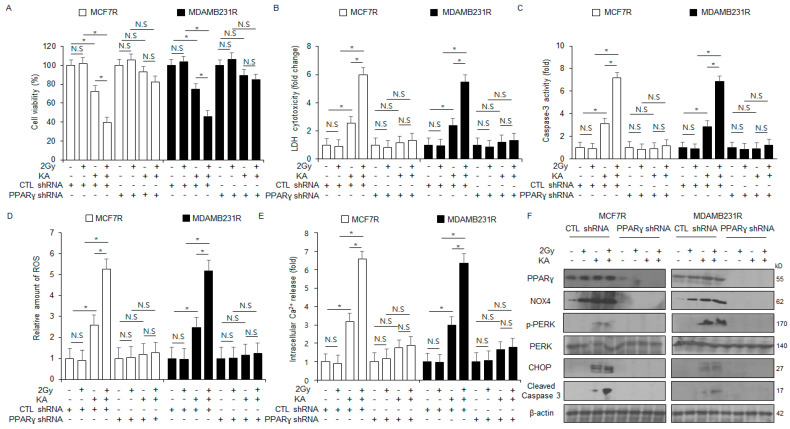
PPARγ silencing inhibits KA + radiation-induced apoptosis in MDA-MB-231R and MCF-7R cells. (**A**–**F**) MDA-MB-231R and MCF-7R cells were treated with PPAR**γ** shRNA particles for gene knockdown. These cells were treated with 100 µM KA and 2 Gy radiation for 24 h and subjected to LDH cytotoxicity, intracellular Ca^2+^ and ROS, caspase-3 activity, and WST-1 assays. Western blot analysis was conducted to examine the protein levels of cleaved caspase-3, PERK, CHOP, PPARγ, NOX4, and p-PERK; * *p* < 0.05. N.S, no significance. β-actin was used as the loading control.

Next, we assessed whether 2 Gy radiation combined with KA treatment mediates ER stress–induced apoptosis via PPARγ in radioresistant MCF-7R and MDA-MB-231R cells. These cells were transfected with PPARγ shRNA for gene silencing, followed by puromycin selection to ensure stable knockdown. In non-transfected MCF-7R and MDA-MB-231R cells, KA treatment alone increased caspase-3 activity, cytotoxicity, and intracellular ROS, and Ca^2+^ levels in these cells. When the cells were co-treated with KA and 2 Gy radiation, these properties were further enhanced; 2 Gy radiation treatment alone did not induce significant changes. Notably, upon PPARγ silencing, none of the treatment groups (KA, 2 Gy, and KA + 2 Gy) induced significant changes in the respective properties (Figure 8A–E). Western blot analysis indicated that KA treatment enhanced the protein levels of PPARγ, CHOP, p-PERK, NOX4, and cleaved caspase-3, with 2 Gy radiation alone inducing no significant changes. The KA + 2 Gy group showed further enhanced levels of these proteins compared to the KA group (Figure 8F). In PPARγ-knockdown MCF-7R and MDA-MB-231R cells, only the KA group showed reduced levels of PPARγ; the 2 Gy and KA + 2 Gy groups did not induce significant changes. These findings demonstrated that KA-induced enhancement in PPARγ expression promotes apoptosis through ROS and ER stress induction. This suggests that KA has the potential to enhance the sensitivity of radioresistant breast cancer cells to radiotherapy.

## 4. Discussion

Recent studies have shown that natural compounds such as flavonoids, alkaloids, phenols, and terpenoids display potent anticancer effects [35,36]. In cancer cells, these compounds often promote intracellular Ca^2+^ and ROS production, thereby inducing oxidative stress–driven apoptosis [37,38]. In this study, we explored KA’s antitumor effects and ability to sensitize radioresistant breast cancer cells to radiotherapy. Our findings demonstrated that KA’s anticancer effects promote apoptosis in breast cancer cells both in vitro and in vivo, highlighting its clinical potential to inhibit tumor growth [39]. By co-treating Z-VAD-FMK and KA, we demonstrated that KA-induced apoptosis in breast cancer cells is dependent on caspase activity. Furthermore, KA enhanced ER stress in these cells, thereby activating the downstream ER stress transmembrane sensors, IRE1α, ATF6, and PERK, inducing apoptosis [40]. Upon UPR activation, the ER chaperone protein GRP78 dissociated from these sensors mediated the phosphorylation of PERK and eIF2α [41]. It has been demonstrated that p-eIF2α induces the upregulation of cytosolic ATF4 and ATF3. The translocated nuclear ATF4 binds to the promoter of CHOP and upregulates its expression [42]. KA was found to mediate the ER stress signaling pathway by promoting the production of intracellular Ca^2+^ and ROS. This phenomenon ultimately induced apoptosis via the PERK-ATF4-CHOP signaling pathway. Silencing PERK or CHOP in these cells led to the suppression of KA-induced apoptosis. When KA was co-treated with TG, synergistic apoptosis was induced via the PERK-ATF4-CHOP axis in breast cancer cells. In this process, NOX4 was identified as a regulator of ROS generation promoted by KA treatment. The activation of NOX4 was found to mediate ER stress–induced apoptosis by promoting intracellular ROS and Ca^2+^ production in KA-treated cells. NOX4 silencing or antioxidant (NAC or DPI) treatment reduced the enhancement in intracellular ROS, caspase-3 activity, cytotoxicity, and the PERK-ATF4-CHOP signaling pathway in KA-treated breast cancer cells. Although radiotherapy is a primary cancer treatment option, tumor cells often develop radioresistance after exposure, reducing the effectiveness of therapy [43]. Recent studies have reported that natural compounds such as curcumin, papaverine, paclitaxel, and genistein are effective radiotherapy sensitizers with minimal side effects [44,45]. The EMT markers have been associated with the development of radioresistance, chemoresistance, and hypoxic environments [46]. The development of anticancer agents that can sensitize radioresistant cancer cells has the potential to enhance the effectiveness of radiotherapy. When radiation was combined with KA, the radioresistant MDA-MB-231R and MCF-7R cells were effectively sensitized to radiotherapy. This phenomenon was indeed demonstrated to be dependent on the modulation of EMT markers, such as vimentin, N-cadherin, and E-cadherin.

Many natural compounds have a potential anticancer efficacy in many cancers [47]. Paclitaxel has been shown to induce apoptosis by promoting the cleavage of caspase-3 and -9 cleavage and decreasing BCL2 expression in human osteogenic sarcoma cells [48]. The sesquiterpene compound arnicolide D has been found to promote ER stress–induced apoptosis via the PERK-ATF4-CHOP axis in hepatocellular carcinoma cells, HepG2 and Huh7, where NAC treatment could inhibit arnicolide D-induced ER stress and apoptosis [49]. Furthermore, rhamnetin and cirsiliol have been demonstrated to display anticancer effects and the ability to sensitize radioresistant cells through the inhibition of EMT markers, including fibronectin, vimentin, and E-cadherin, in non-small cell lung cancer cells [50]. Analogously to these natural compounds, we demonstrated that KA promote apoptosis in breast cancer cells via the PERK-ATF4-CHOP axis. Importantly, KA was found to effectively sensitize radioresistant cells by modulating the aforementioned EMT markers. Recently, it has been demonstrated that KA induces caspase-3–driven apoptosis by downregulating miR-21 in human malignant glioblastoma cells [51].

ROS release and mitochondrial dysfunction promote ER stress and apoptosis by upregulating ATF4 and CHOP in various cancer types [52]. Tocotrienol has been shown to induce apoptosis in MCF-7 and MDA-MB-231 cells via the CHOP-DR5 axis [53]. NOXs are enzymes that generate ROS and serve as potential regulators of cancer cell metabolism [54]. The seven transmembrane NOXs, including dual oxidases 1 and 2 and NOX1-5, produce superoxide anion radicals and play various roles in pathophysiological disorders, including cardiovascular diseases, inflammation, neurodegeneration, and cancer [55]. In the mitochondria, NOX4 activity is regulated by adenosine triphosphate (ATP) and plays a crucial role in modulating cell survival and death [56]. Furanodienone has been found to promote NOX4-induced generation of mitochondrial ROS and induce caspase-dependent apoptosis through the PRDX1-MAPKs-p53 axis in colorectal cancer cells [57]. Similarly, we demonstrated that KA induces apoptosis via the PERK-ATF4-CHOP pathway driven by NOX4-induced generation of ROS. Silencing NOX4 or antioxidant (DPI or NAC) treatment reduced the enhancements in intracellular ROS, cytotoxicity, and caspase-3 activity in KA-treated breast cancer cells, indicating NOX4 as the regulator of KA-induced ROS generation.

Radiotherapy utilizes high-energy beams to destroy cancer cells, but breast cancer patients often develop radioresistance [58]. Therefore, strategies to sensitize resistant cancer cells to radiation are critically needed [59]. Radiation exposure reduces oxygen levels in TME, leading to a hypoxic state that induces radioresistance by promoting EMT [60]. In this process, the epithelial cell regulatory protein E-cadherin is downregulated, while mesenchymal cell regulatory proteins such as vimentin and N-cadherin are upregulated [61]. In radioresistant MDA-MB-231R and MCF-7R cells, KA combined with radiation inhibited these trends, inhibiting radioresistance.

Accumulating evidence suggests that synthetic and natural PPARγ ligands exert therapeutic effects by modulating immune responses, lipid metabolism, cancer cell apoptosis, metastasis, and angiogenesis [62]. In breast cancer, the PPARγ ligand pioglitazone has been shown to act as a tumor suppressor via the JAK2/STAT3 signaling axis [63]. Additionally, rosiglitazone has been demonstrated to negatively regulate hypoxia-induced NOX4 expression via NF-kB and promote cell proliferation in human pulmonary artery smooth muscle cells [64]. PPARγ silencing was found to promote cell proliferation by upregulating NOX4 in these cells [65]. However, the relationship between PPARγ and NOX4 for PPARγ ligands with anticancer effects remains incompletely understood. Our findings suggested that KA-induced enhancement in PPARγ activity led to ER stress and apoptosis, which subsequently led to the upregulation of NOX4 in the treated cancer cells. PPARγ silencing effectively suppressed the observed apoptosis in KA-treated cells by inhibiting NOX4 and the PERK-ATF4-CHOP signaling pathway. Ultimately, KA combined with radiation significantly increased ER stress and apoptosis by modulating EMT markers in radioresistant breast cancer cells

## 5. Conclusions

In conclusion, we investigated and validated KA’s anticancer and anti-inflammatory properties both in vitro and in vivo. KA treatment was shown to enhance PPARγ activity and mediates apoptosis via the PERK-CHOP signaling pathway. Furthermore, the treatment enhanced the levels of cytosolic Ca^2+^ and ROS and upregulated NOX4 in breast cancer cells. When KA was combined with radiation, radioresistance was effectively overcome through EMT modulation in MCF-7R and MDA-MB-231R cells.

## Data Availability

Data are contained within the article.
